# Postdischarge-to-30-Day Mortality Among Patients Receiving MitraClip: A Systematic Review and Meta-Analysis

**DOI:** 10.1016/j.shj.2022.100011

**Published:** 2022-04-26

**Authors:** Beni R. Verma, Shashank Shekhar, Toshiaki Isogai, Raghuram Chava, Pejman Raeisi-Giglou, Agam Bansal, Shameer Khubber, Bryce Montane, Prashansha Vaidya, Simrat Kaur, Manpreet Kaur, Rhonda Miyasaka, Serge C. Harb, Amar Krishnaswamy, Samir R. Kapadia

**Affiliations:** a Heart and Vascular Institute, Cleveland Clinic, Cleveland, Ohio, USA; b Department of Cardiology, Case Western MetroHealth Health System, Cleveland, Ohio, USA; c Department of Surgery, Cleveland Clinic, Cleveland, Ohio, USA

**Keywords:** Heart failure, Mitral valve disease, Mortality, Percutaneous valve therapy, Structural heart disease intervention

## Abstract

**Background:**

MitraClip (MC) implantation is the recommended treatment for severe symptomatic mitral regurgitation in patients not responding to medical therapy and at prohibitive surgical risk. It is important to quantify immediate mortality during postdischarge-to-30-day period so as to improve the procedural outcomes. Hence, we aim to identify the incidence of postdischarge-to-30-day mortality and its associated predictors using the technique of meta-analysis.

**Methods:**

We searched Medline, Embase, and Cochrane CENTRAL databases from inception until July 3, 2019 for studies reporting mortality prior to discharge, at 30 days and 1 year after MC implantation. The primary outcome was postdischarge-to-30-day all-cause mortality.

**Results:**

Of 2394 references, 15 studies enrolling 7498 patients were included. Random effects analysis showed that all-cause cumulative inpatient, 30-day, and 1-year mortality was 2.40% (2.08, 2.77; *I*^*2*^ = 0%), 4.31% (3.64, 5.09, *I*^*2*^ = 41.9%), and 20.71% (18.32; 23.33, *I*^*2*^ = 81.5%), respectively. The postdischarge-to-30-day mortality was 1.70% (95% confidence interval: 1.0, 2.70; *I*^*2*^ = 84%). A total of 71.50% of deaths (95% confidence interval: 36.80-91.50, *I*^*2*^ = 63%) in the postdischarge-to-30-day period were due to cardiac etiology. On meta-regression, pre-MC left ventricular ejection fraction (*p* = 0.003), Log.Euroscore (*p* = 0.047), Society of Thoracic Surgeons Predicted Risk of Mortality (*p* < 0.001), and prolonged ventilation >48 ​hours (*p* < 0.001) were found to be its significant predictors.

**Conclusions:**

Our meta-analysis reports an additional mortality of ∼2% immediately after MC implantation during the postdischarge-to-30-day period. Majority of deaths occurred due to cardiac causes. Pre-MC left ventricular ejection fraction, Log.Euroscore, Society of Thoracic Surgeons Predicted Risk of Mortality score, and prolonged ventilation were found to be its significant predictors. Further studies are needed to better understand the causes of this early mortality to maximize benefits of this important therapy.

## Introduction

Transcatheter mitral valve repair with MitraClip (MC) implantation is the recommended treatment for patients with severely symptomatic mitral regurgitation (MR) and high-risk surgical candidacy.^1^ Procedurally, it involves edge-to-edge approximation of anterior and posterior mitral valve leaflets using a metallic clip.^2^ It has been proven to be a safe and an efficacious treatment leading to improvement in the quality of life, degree of MR, and heart failure (HF) symptoms.^3^, ^4^, ^5^, ^6^ MC is usually performed under general anesthesia with the help of fluoroscopy and transesophageal echocardiography. Perioperative anesthesia requirements are complex and resemble those for cardiac surgery, which is a testament to patient complexity and the procedure itself.^7^^,^^8^

The reported in-hospital and cumulative 30-day mortality rate after MC implantation is 0-4% and 5.8%, respectively,^6^^,^^9^ which attests to the safety of the procedure in a high-risk population. Patient comorbidities such as advanced age, history of advanced HF, atrial fibrillation (AF), renal failure, or lung disease are identified as predictors of increased mortality.^4^^,^^10^^,^^11^

In the Cardiovascular Outcomes Assessment of the MitraClip Percutaneous Therapy for Heart Failure Patients with Functional Mitral Regurgitation (COAPT) study, the 30-day mortality was 2.3%. In the Multicenter Study of Percutaneous Mitral Valve Repair MitraClip Device in Patients with Severe Secondary Mitral Regurgitation (MITRA-FR) trial, patients receiving MC experienced 0% periprocedural mortality up to 3 days after the procedure, whereas the cumulative 30-day mortality was 3.3%.^12^^,^^13^ In these studies, mortality prior to discharge and from discharge to 30 days were not reported separately. Taken together, these observations suggest mortality risk from MC in the early postprocedural period is distinct from intraprocedural risk with an unclear mechanism. We, therefore, conducted this systematic review and meta-analysis to quantify this risk and study the predictors of postdischarge-to-30-day mortality among patients receiving MC.

## Methods

Our systematic review is designed to address the following questions: What is the incidence of postdischarge-to-30-day mortality among patients receiving MC and what are its associated predictors? Our study population is defined as patients with moderate-to-severe MR, study intervention is defined as MC implantation, and outcomes (primary) include mortality during the postdischarge-to-30-day period. Mortality at discharge and after discharge within 1 year, HF readmission rates at 30 days and 1 year, cardiac deaths, reintervention with MC, and mitral valve surgery after discharge are reported as secondary outcomes. Time to event at discharge, from discharge to 30 days, and from discharge to 1 ​year is indexed from MC implantation.

We screened articles based on the population, intervention, comparison, and outcomes framework for randomized controlled trials and observational and cohort studies. Pediatric or animal studies, letters to the editor, review articles, case presentations, meeting abstracts, commentaries, and opinion articles were not included. We also excluded studies which lacked the primary outcome at discharge, 30 days, or 1 year, conducted <100 MC procedures (to avoid bias of selective reporting of outcomes), and reported secondary analysis of MC registries, i.e., multiple papers from one data source (to avoid overlap of study population). Our study did not involve any direct interaction with human or animal subjects by any of the authors and thus did not meet criteria to require institutional review board approval.

Ovid MEDLINE, Embase, Cochrane Central Register of Controlled Trials, Web of Science, and other sources (such as google scholar) database were searched from inception till July 3, 2019. Our search terms included the following: “mitral valve regurgitation” OR “mitral valve insufficiency” OR “mitral regurgitation” OR “mitral insufficiency” OR “degenerative mitral valve disease” OR “degenerative MR” OR “DMR” OR “functional mitral regurgitation” OR “FMR” OR “myxomatous mitral valve disease” OR “mitral annular calcification” OR “MAC” OR “mitral annular fibrosis” OR “mitral valve prolapse” OR “mitral valve prolapse” OR “MVP” OR “mitral valve tethering” AND “mitral clip” OR “MC” OR “mitral clipping” OR “mitral valve clip” OR “mitral clip system” OR “edge-to-edge mitral valve repair” OR “percutaneous mitral valve repair” OR “PMVR” OR “transcatheter mitral valve repair” OR “TMVR” OR “transcatheter mitral valve intervention” OR “percutaneous mitral annuloplasty” AND “mortality”.

Studies were screened by 2 independent operators (BV and SS) for selection criteria, as described earlier. Studies were reviewed manually to extract the required data for qualitative and quantitative analysis including meta-proportion and meta-regression. Selected articles were thoroughly discussed by 2 reviewers (BV and SS), and in case of any conflict, mutual approval was obtained. All data were obtained from the manuscript text, figures, and tables. Kaplan-Meier estimates from these studies were examined to extract number of deaths at 30 days and 1 year. The number of deaths was computed by deriving the cumulative mortality rate at 30 days and 1 ​year from Kaplan-Meier curves and then multiplying it with the total number of study patients. Postdischarge-to-30-day mortality was calculated by subtracting deaths due to all causes prior to discharge from the cumulative all-cause 30-day mortality. Similarly, postdischarge-to-1-year mortality was calculated as the difference of cumulative 1-year mortality and deaths prior to discharge. The Newcastle-Ottawa Quality Assessment Scale (Appendix A) was used to evaluate the quality of evidence among the included studies.

Meta-Analysis technique was used to derive the incidence of postdischarge-to-30-day mortality as well as deaths prior to discharge and postdischarge-to-1-year deaths. The inverse-variance method (DerSimonian-Laird estimator) along with logit transformation for meta-proportion, continuity correction of 0.5 in studies with 0 ​cell frequencies, and Clopper-Pearson for calculation of the confidence interval was applied.^14^, ^15^, ^16^ This method was preferred over Mantel-Haenzel since the effect size was the proportion of an event rather than an odds ratio. The random effects model was used for reporting results. Calculated meta-proportion was represented per 100 MC procedures. Heterogeneity with *I*^*2*^ > 50% was defined as significant.

Meta-Regression using the mixed effects model was performed to identify predictors of postdischarge-to-30-day mortality. Variables such as age, male gender, hypertension, cerebrovascular accidents, diabetes mellitus, coronary artery disease, myocardial infarction, coronary artery bypass grafting, AF, congestive HF (CHF), chronic kidney disease (CKD), chronic obstructive lung disease, body mass index (kg/m^2^), peripheral artery disease, pro-brain natriuretic peptide, single vs. multicenter study, prospective vs. retrospective nature of the study cohort, pre-MC left ventricular ejection fraction (LVEF), pre-MC severe tricuspid regurgitation, procedural success, Society of Thoracic Surgeons score, Log.Euroscore, post-MC MR >3, number of mitral clips implanted >1, clip detachment, prolonged ventilation >48 ​hours, post-MC bleeding, and post-MC conversion to open-heart surgery were studied. A *p*-value <0.05 was considered statistically significant. Publication bias or small study effect was assessed via visual inspection of the funnel plot and linear regression test of funnel plot asymmetry. Sensitivity analysis was designed to evaluate robustness of our calculated primary outcome by omitting studies contributing >25% weight (random) to our meta-analysis. Statistical software RStudio (Version 1.3.1093 – © 2009-2020 RStudio, PBC Boston, MA; packages—meta, metafor, metagen, and rmeta) was used for our analysis and production of figures.

## Results

**Figure 1:**
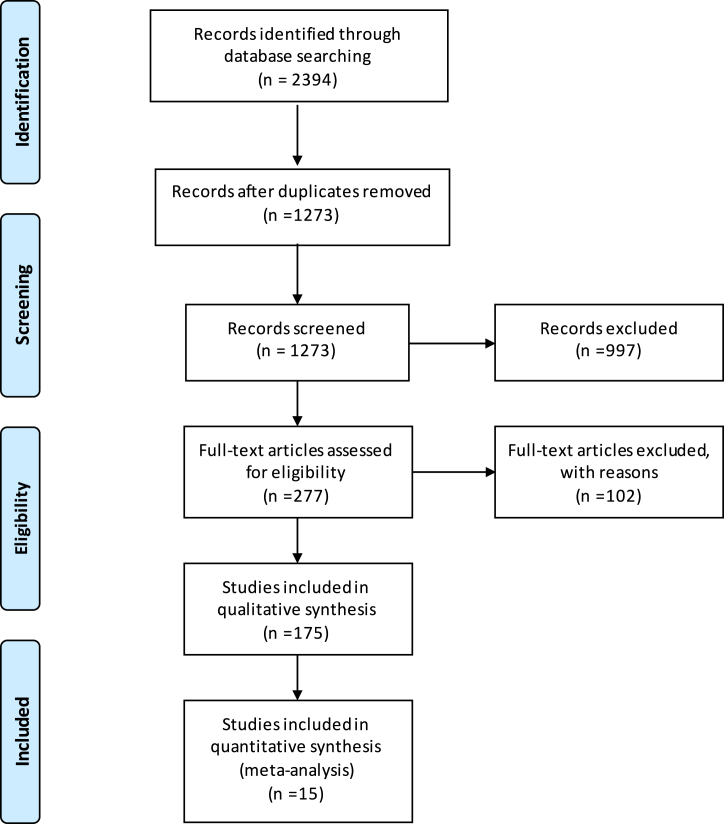
**PRISMA flow diagram summarizing search strategy to identify studies for postdischarge-to-30-day mortality among patients with MitraClip**.

A total of 2394 records were identified. A total of 1121 records were removed during the process of deduplication. A total of 1273 records were included for abstract and title screening. We excluded 997 articles as they were (a) not relevant to our research question, (b) review articles, (c) case reports/abstracts/supplements or letters to the editor, (d) animal studies, and (e) pediatric studies. Two hundred seventy-seven records were assessed for detailed manuscript review using the previously mentioned population, intervention, comparison, and outcomes framework. Of 277 records, 175 records reported pertinent information, of which 15 studies met our selection criteria (Figure 1).

Of these 15 studies, 47% (7/15) of studies were prospective,^5^^,^^6^^,^^17^, ^18^, ^19^, ^20^, ^21^ and 53% (8/15) were retrospective.^10^^,^^11^^,^^22^, ^23^, ^24^, ^25^, ^26^, ^27^ Forty-three percent (3/7) of the prospective studies^5^^,^^6^^,^^17^ and 63% (5/8) of retrospective studies^11^^,^^22^^,^^24^^,^^26^^,^^27^ were conducted at multicenter locations. Retrospective studies contributed 5123 patients and prospective studies added 2375 patients toward a total of 7498 MC patients for our analysis (Table 1). The mean age of these patients from included studies was 75 years. Patients included in these studies were high-risk surgical candidates with median Log.Euroscore II of 23, median procedure time of 130 ​minutes, and length of stay of 6 days. Baseline characteristics of study patients are provided in Table 2.

**Table 1:** Study characteristics

Authors et al. (year of publication)	Registry	Center	Study design	Patient enrollment period	Number of study patients∗
Ailawadi et al. (2019)^17^	EVEREST II	Multicenter	Prospective	August 2005 till December 2013	616
Arora et al. (2019)^11^	TVT	Multicenter	Retrospective	November 2013 till June 2016	5613
Kitamura et al. (2019)^22^	Institutional	Multicenter	Retrospective	September 2009 till June 2016	341
Kreusser et al. (2019)^23^	Institutional	Single-center	Retrospective	September 2009 till January 2016	174
Geis et al. (2017)^24^	TRAMI	Multicenter	Retrospective	August 2010 till August 2013	777
Osteresch et al. (2018)^25^	Institutional	Single-center	Retrospective	March 2013 till April 2015	130
Metze et al. (2017)^18^	Institutional	Single-center	Prospective	May 2014 till June 2016	213
Saji et al. (2017)^26^	Institutional	Multicenter	Retrospective	March 2007 till February 2016	222
Giannini et al. (2016)^27^	Institutional	Multicenter	Retrospective	October 2008 till October 2015	169
Oner et al. (2016)^19^	Institutional	Single-center	Prospective	February 2010 till September 2013	165
Schau et al. (2016)^10^	Institutional	Single-center	Retrospective	March 2009 till May 2013	194
Bozdag-Turan et al. (2014)^20^	Institutional	Single-center	Prospective	February 2010 till July 2012	121
Nickenig et al. (2014)^6^	Transcatheter Valve Treatment Sentinel Pilot Registry	Multicenter	Prospective	January 2011 till December 2012	628
Grasso et al. (2013)^21^	GRASP	Single-center	Prospective	August 2008 till October 2012	117
Maisano et al. (2013)^5^	ACCESS-EU	Multicenter	Prospective	April 2009 till April 2011	567

Abbreviations: ACCESS-EU, MitraClip Therapy Economic and Clinical Outcomes Study Europe; EVEREST II, Endovascular Valve Edge-to-Edge Repair Study II; GRASP, Getting Reduction of Mitral Insufficiency by Percutaneous Clip Implantation; TRAMI, transcatheter mitral valve intervention; TVT, transcatheter valve therapy.

∗ Represents number of study patients receiving MitraClip. To calculate postdischarge-to-30-d mortality, we only included patients who were discharged alive.

**Table 2:** Patient characteristics

Authors et al. (year of publication)	Number of study patients	Age	Male	Hypertension	DM-2	MI	CAD	AFIB	CABG	CHF	CVA	COPD	Obesity	CRF	PAD	LVEF	STS PROM score	Log.EUROSCORE II	Type of MR	NT-pro BNP (pg/mL)
Ailawadi et al. (2019)^17^	616	73.3	364 (59)	546 (89)	240 (39)	342 (56)	498 (81)	400 (65)	342 (56)	602 (98)	136 (22)	192 (31)	NA	163 (27)	120 (20)	43.2	10.2	NA	FMR	NA
Arora et al. (2019)^11^	5613	79	2997 (53)	4765 (85)	1482 (26)	1555 (28)	NA	3536 (63)	1656 (30)	4715 (84)	595 (11)	NA	NA	3244 (58)	1038 (19)	28.9	NA	NA	NA	NA
Kitamura et al. (2019)^22^	341	75	260 (76)	126 (79)	143 (43)	180 (53)	NA	208 (61)	NA	317 (93)	NA	217 (23)	78 (26)	NA	75 (22)	30.5	NA	26.5	FMR	4200
Kreusser et al. (2019)^23^	174	75.2	121 (70)	131 (75)	51 (29)	NA	100 (58)	63 (36)	NA	174 (100)	24 (14)	33 (19)	NA	NA	NA	25	14.7	24.4	BOTH	4504
Geis et al. (2017)^24^	777	75.8	469 (60)	NA	243 (31)	213 (27)	613 (79)	NA	NA	691 (89)	78 (10)	177 (23)	198 (26)	NA	NA	NA	NA	20	BOTH	3568
Osteresch et al. (2018)^25^	130	72.7	83 (64)	98 (75)	34 (26)	45 (35)	77 (59)	51 (39)	35 (27)	130 (100)	NA	21 (16)	NA	NA	NA	32	NA	23.8	FMR	9140
Metze et al. (2017)^18^	213	77	121 (57)	NA	60 (28)	66 (31)	126 (59)	121 (57)	NA	NA	28 (13)	NA	55 (26)	NA	NA	NA	NA	17	BOTH	NA
Saji et al. (2017)^26^	222	77.3	110 (50)	166 (75)	60 (27)	70 (32)	138 (62)	138 (62)	NA	217 (98)	36 (16)	71 (32)	56 (25)	13 (6)	25 (11)	46.9	NA	NA	BOTH	NA
Giannini et al. (2016)^27^	169	72.1	132 (78)	115 (68)	53(31)	93 (55)	108 (64)	68 (40)	51 (30)	130 (77)	6 (4)	46 (27)	42 (25)	47 (28)	NA	31	NA	19.6	FMR	3458
Oner et al. (2016)^19^	165	78	92 (56)	154 (93)	70 (42)	NA	NA	106 (64)	NA	153 (93)	23 (14)	51 (31)	43 (26)	89 (54)	NA	41	10.8	NA	BOTH	NA
Schau et al. (2016)^10^	194	74	124 (64)	NA	66 (34)	NA	122 (63)	NA	NA	194 (100)	NA	NA	NA	NA	NA	41	NA	23	BOTH	3452
Bozdag-Turan et al. (2014)^20^	121	77	69 (57)	113 (93)	48 (40)	NA	NA	80 (66)	NA	110 (91)	16 (13)	34 (28)	32 (26)	66 (55)	NA	42	10.9	NA	BOTH	NA
Nickenig et al. (2014)^6^	628	74.2	396 (63)	477 (76)	175 (28)	196 (31)	194 (31)	199 (32)	203 (32)	537 (86)	90 (14)	121 (19)	NA	192 (31)	NA	42.6	NA	NA	BOTH	NA
Grasso et al. (2013)^21^	117	72	78 (67)	92 (79)	40 (34)	33 (28)	57 (49)	47 (40)	22 (19)	94 (80)	8 (7)	25 (21)	NA	45 (38)	NA	38	NA	12	BOTH	NA
Maisano et al. (2013)^5^	567	73.7	362 (64)	432 (76)	168 (30)	181 (32)	356 (63)	384 (68)	164 (29)	397 (70)	73 (13)	108 (19)	NA	236 (42)	NA	NA	NA	23	BOTH	NA

*Notes*. Variables are represented as N (%) or mean; age is represented in years (mean).

Abbreviations: AFIB, atrial fibrillation; CABG, coronary artery bypass grafting; CAD, coronary artery disease; CHF, congestive heart failure; COPD, chronic obstructive pulmonary disease; CRF, chronic renal failure; CVA, cerebrovascular accident; DM-2, diabetes mellitus type 2; DMR, degenerative MR; FMR, functional MR; LVEF, left ventricular ejection fraction (expressed in %); MI, myocardial infarction; MR, mitral regurgitation; NA, not available; NT-proBNP, N-terminal pro b-type natriuretic peptide; PAD, peripheral arterial disease; STS PROM, Society of Thoracic Surgery Predicted Risk of Operative Mortality score.

**Figure 2:**
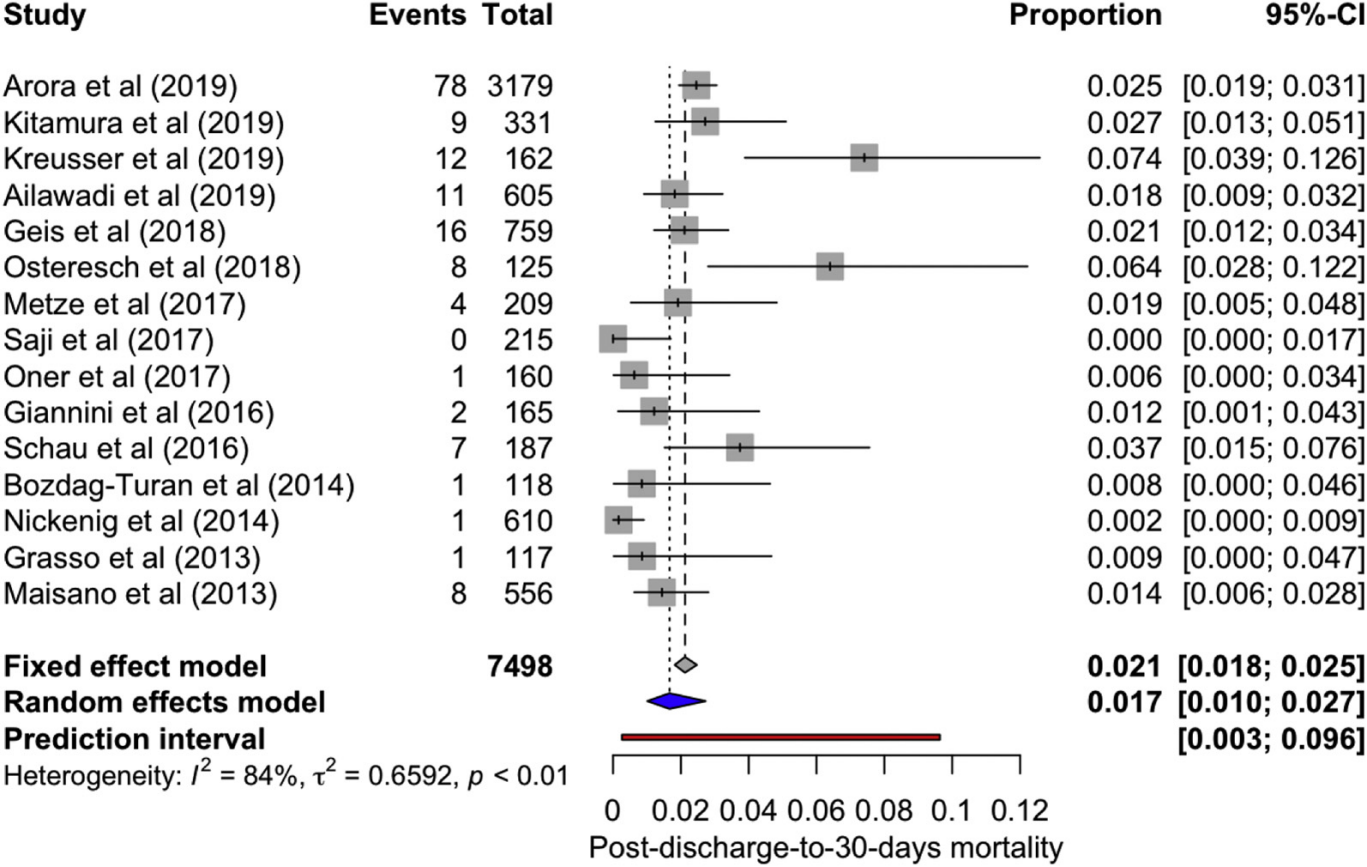
**Forest plot showing individual and summary proportion of all-cause postdischarge-to-30-day mortality among patients receiving MitraClip as 1.70% [95% CI: 1.0-2.70; *I***^***2***^**= 84%].** Abbreviation: CI, confidence interval.

**Figure 3:**
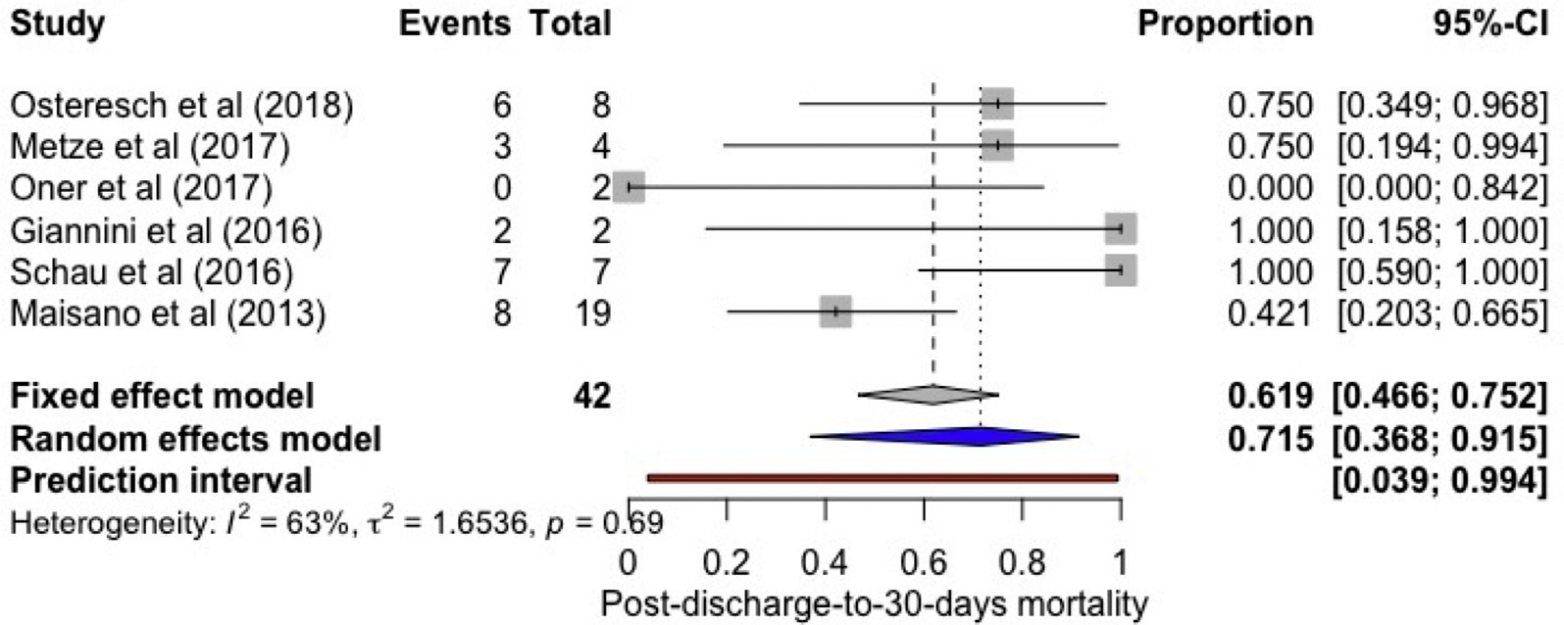
**Forest plot showing individual and summary proportion of postdischarge-to-30-day mortality due to cardiac etiology among patients receiving MitraClip as 71.50% [95% CI: 36.80-91.50, *I***^***2***^**= 63%].** Abbreviation: CI, confidence interval.

All-cause inpatient, cumulative 30-day, and 1-year mortality was 2.40% (2.08, 2.77; *I*^*2*^ = 0%), 4.31% (3.64, 5.09, *I*^*2*^ = 41.9%), and 20.71% (18.32; 23.33, *I*^*2*^ = 81.5%), respectively. The incidence of postdischarge-to-30-day all-cause mortality was found to be 1.70% (95% confidence interval [CI]: 1.0, 2.70; *I*^*2*^ = 84%) (Figure 2). A total of 71.50% (95% CI: 36.80-91.50, *I*^*2*^ = 63%) of postdischarge-to-30-day deaths were due to cardiac causes (Figure 3).

**Figure 4:**
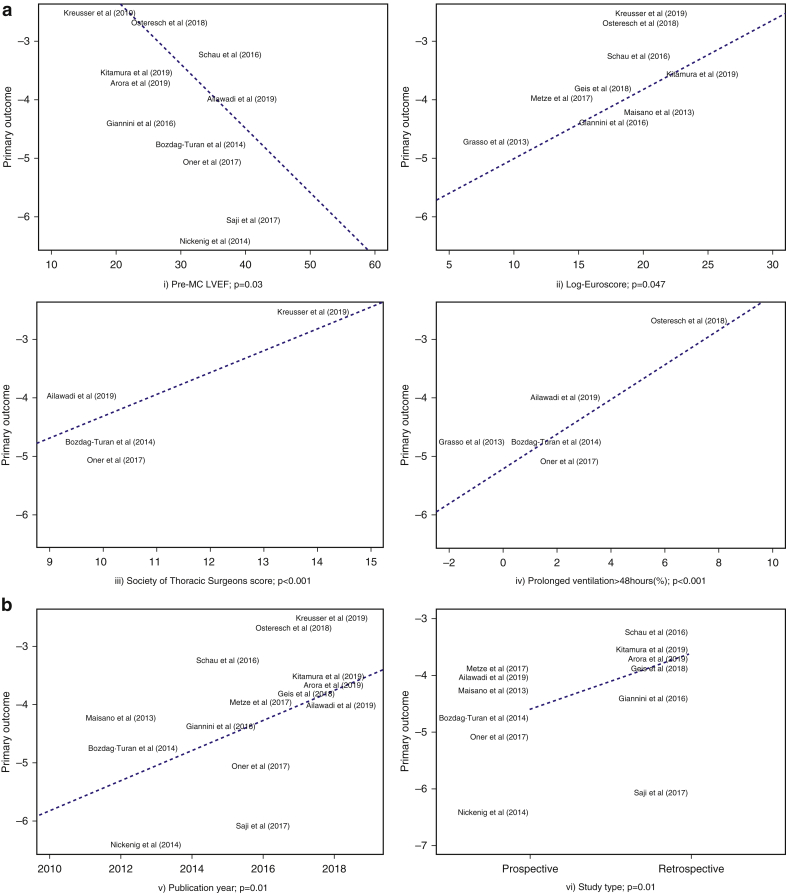
(a) Meta regression—(i) pre-MC left ventricular ejection fraction (*p* = 0.003), (ii) Log.Euroscore (*p* = 0.047), (iii) Society of Thoracic Surgeons score (*p* < 0.001), and (iv) prolonged ventilation >48 ​hours (%) (*p* < 0.001) are found to be statistically significant predictors of all-cause postdischarge-to-30-day mortality; (b) Meta regression—(v) publication year (*p* = 0.01) and (vi) study type (*p* = 0.01) are found to be statistically significant predictors of all-cause postdischarge-to-30-day mortality. Abbreviations: LVEF, left ventricular ejection fraction; MC, MitraClip.

Preprocedure LVEF (*p* = 0.003), Log.Euroscore (*p* = 0.047), Society of Thoracic Surgeons Predicted Risk of Operative Mortality score (STS PROM) (*p* < 0.001), prolonged ventilation >48 ​hours (*p* < 0.001), nature of the study cohort (*p* = 0.01), and publication year (*p* = 0.010) were found to be significant predictors of postdischarge-to-30-day all-cause mortality on meta-regression (Figure 4). A trend is noticed toward higher number of reported outcomes from the studies published in recent years via visual inspection of forest plots and meta-regression analysis. The remaining characteristics, as mentioned earlier in the methods section, including procedural success (*p* = 0.62), failed to reach statistical significance (Appendix B).

**Figure 5:**
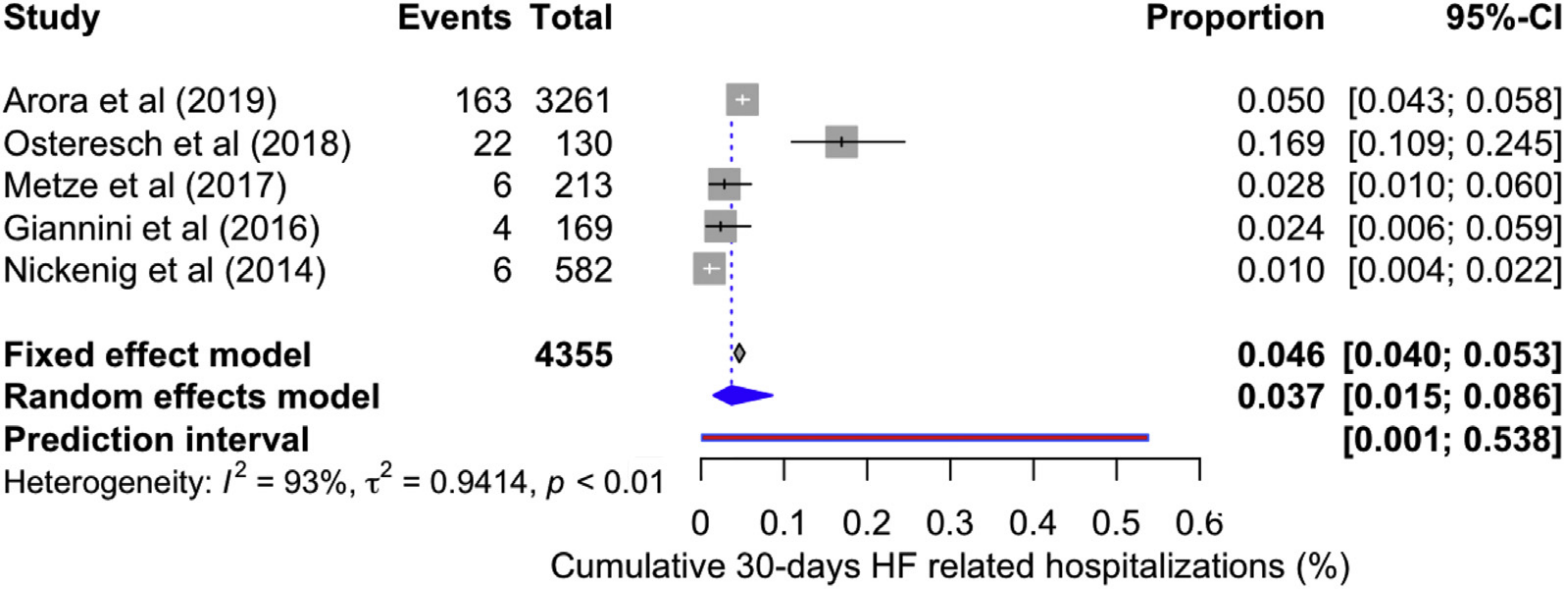
**Forest plot showing individual and summary proportion of cumulative heart failure–related hospitalization at 30 days as 3.70% [95% CI: 1.50-8.60, *I***^***2***^**= 93%].** Abbreviations: CI, confidence interval; HF, heart failure.

The rate of cumulative CHF rehospitalizations after discharge to 30 days was found to be 3.70% (95% CI: 1.50-8.60, *I*^*2*^ = 93%) (Figure 5). Visual inspection of the funnel plot for postdischarge-to-30-day all-cause (linear regression test for funnel plot asymmetry: *p* = 0.177) and cardiovascular mortality revealed no small study effect (Online Supplement 1a and b). Hence, postdischarge-to-30-day mortality of ∼2% forms a significant portion of cumulative 30-day mortality of 4.31%.

**Figure 6:**
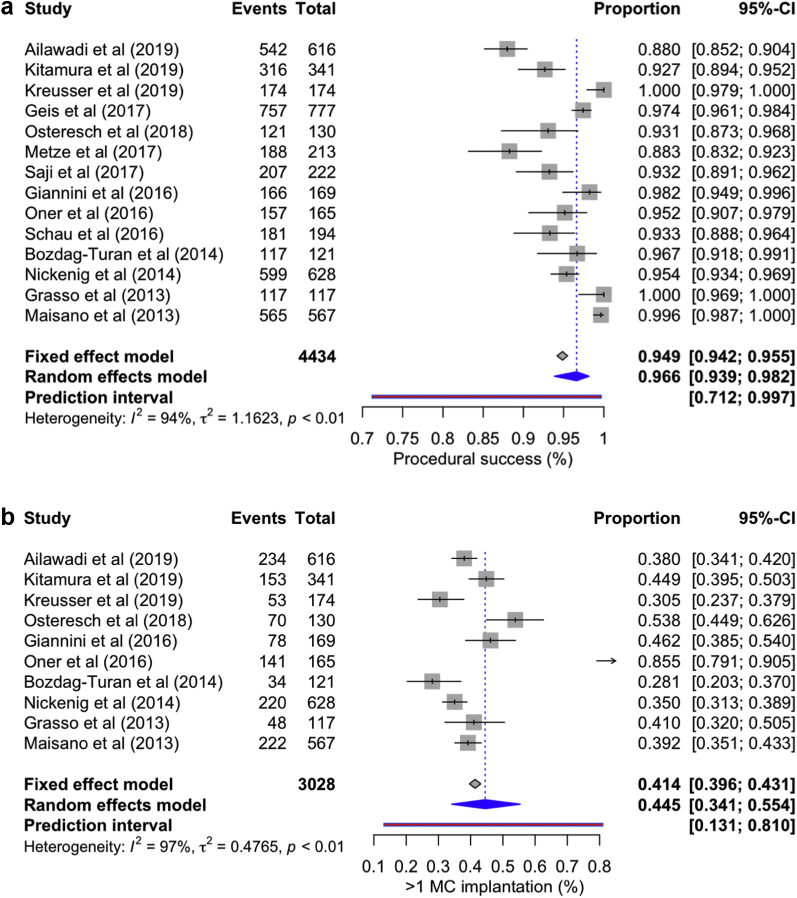

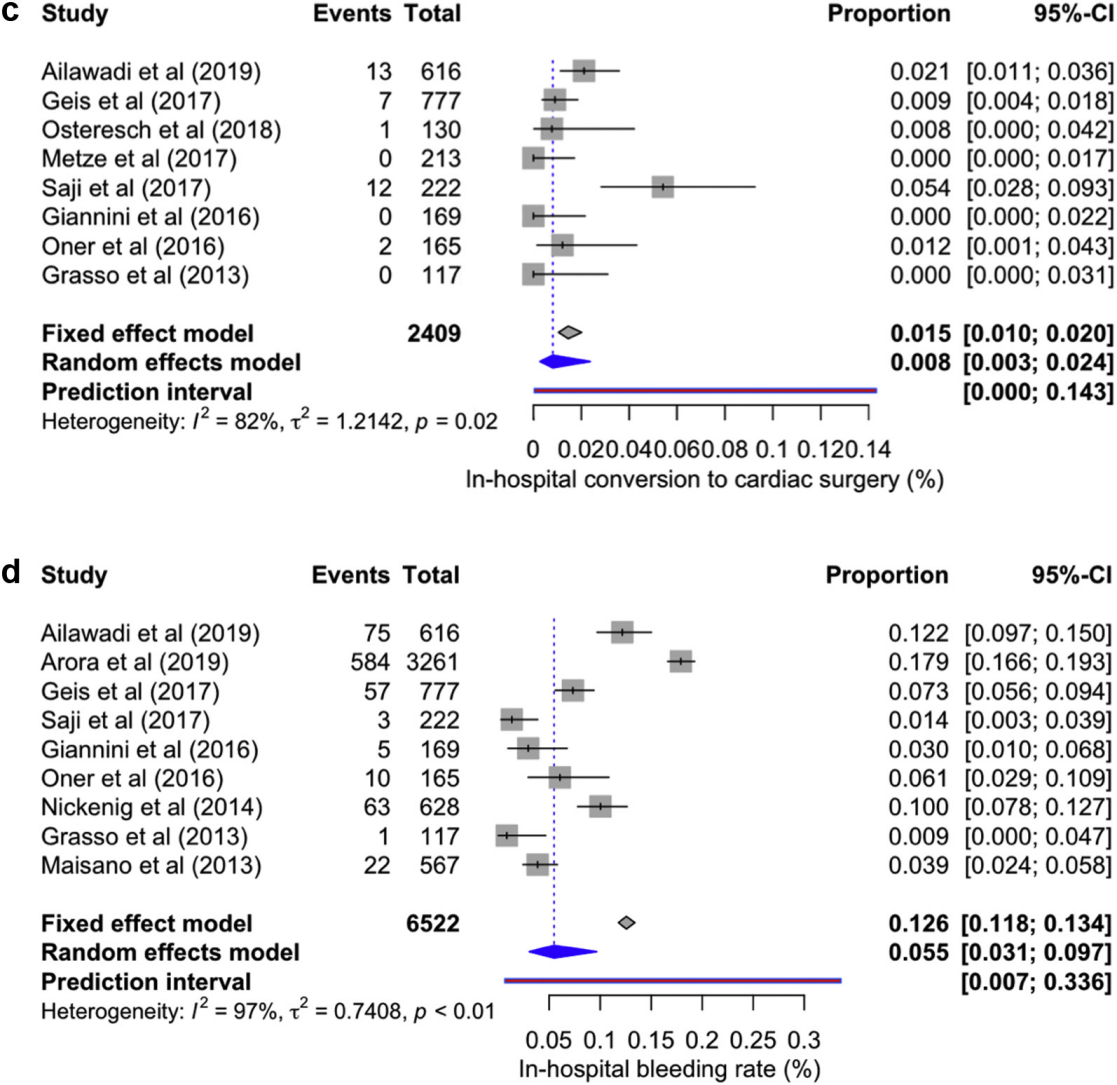
Forest plot showing individual and summary proportion of (a) procedure success—96.60% [95% CI: 93.90-98.20, *I*^*2*^ = 94%]; (b) >1 mitral valve clip implanted in 44.5% [95% CI: 34.10-55.40, *I*^*2*^ = 97%]; (c) in-hospital conversion to open-heart surgery in 0.8% [95% CI: 0.3-2.40, *I*^*2*^ = 82%], and (d) blood transfusion in 5.50% [95% CI: 3.10-9.70, *I*^*2*^ = 97%]. Abbreviations: CI, confidence interval; MC, MitraClip.

Procedure success (post-MC MR grade <2) was attained in 96.60% cases (95% CI: 93.90-98.20, *I*^*2*^ = 94%). >1 mitral valve clip was implanted in 44.5% of patients (95% CI: 34.10-55.40, *I*^*2*^ = 97%); in-hospital conversion to open heart surgery occurred in 0.8% (95% CI: 0.3-2.40, *I*^*2*^ = 812%), and 5.50% [95% CI: 3.10-9.70, *I*^*2*^ = 97%] of patients required blood transfusion (Figure 6a-d). Duration of stay in the intensive care unit ranged from 24 to 40 ​hours; however, only 3.41% (95% CI: 2.79-5.01, *I*^*2*^ = 66%) of patients required prolonged ventilation >48 ​hours. A total of 3.7% (95% CI: 2.9-4.6, *I*^*2*^ = 6%) of patients required MC, and 4.8% (95% CI: 3.02-7.57, *I*^*2*^ = 80.2%) underwent mitral valve surgery after discharge through the follow-up period in the respective studies.

Studies did not report prior-to-discharge mortality among functional and degenerative MR patients. Hence, we compared cumulative 30-day and 1-year mortality between functional and degenerative MR as 0.42 (95% CI: 0.27-0.63; *I*^*2*^ = 0%, *p*-value < 0.0001) and 1.30 (95% CI 0.78-2.12, *I*^*2*^ = 67.4%, *p*-value: 0.32), respectively. Arora et al.^11^ contributed a significant number of patients (n = 3179) to our cohort. It was omitted during sensitivity analysis (to check robustness of our calculated primary outcome) which then revealed the incidence of postdischarge-to-30-day mortality as 1.60% (95% CI: 0.90-2.7, *I*^*2*^ = 80%) (Online Supplement 2). The prior-to-discharge and postdischarge-to-1-year mortality rate was 2.32% (95% CI: 1.92-2.81, *I*^*2*^ = 0%) and 17.64% (95% CI: 15.11-20.50, *I*^*2*^ = 79.7%), respectively.

## Discussion

Our study is the first to focus on the incidence of postdischarge-to-30-day mortality of patients receiving MC. It is interesting to notice that soon after leaving the hospital, 2% of patients die, and the majority of these deaths are attributed to cardiac causes. This constitutes a significant (almost 50%) part of the overall 30-day mortality (4.31%). The mechanism of cardiac decompensation in these patients remains unclear and may represent a potential opportunity to maximize the benefit of this therapy. Pre-MC LVEF, Log.Euroscore, STS PROM score, prolonged ventilation >48 ​hours, nature of the study cohort, and publication year significantly predicted postdischarge-to-30-day mortality.

MC is a safe and viable treatment option for selected patients with severe MR.^28^ The prior-to-discharge mortality from the current literature is 0-4%.^6^^,^^9^ Since it leads to improvement in functional status and quality of life even among frail patients, procedural outcomes such as mortality have become a topic of debate.^18^

It is possible that the procedural complications may be responsible for these deaths during the postdischarge-to-30-day period; however, it is difficult to comment based on the available data. The procedure success rate was found to be not associated with our early mortality, probably due to a consistently high success rate (>95%) across these studies. It is interesting to report that cardiac complications are the most common cause of death during this period. This could be due to worsening MR in some patients who eventually require repeat MC or mitral valve surgery after the index procedure, residual left-to-right shunt causing right-sided decompensation in patients with impaired right ventricular (RV) function or potential right-to-left shunt in patients with high right atrial pressures from RV dysfunction, or tricuspid regurgitation that is not recognized until the patient is ambulatory.^29^, ^30^, ^31^ “Pump failure”—cardiogenic shock—is another possible mechanism of cardiac deaths among these patients.^32^ There may be an opportunity to mitigate these risks if we understand the mechanisms of death in this early postprocedural period.

We noticed that pre-MC LVEF, Log.Euroscore, STS PROM score, and prolonged ventilation are significant predictors of postdischarge-to-30-day mortality. This is a unique finding which can guide clinicians to identify at-risk patients during the postprocedure period. This again points to a sicker patient population with potential worsening of right or left HF as the possible mechanism. Also, studies published in recent years reported higher mortality, which could be attributed to MC procedures being performed in high acuity cases, their widespread utilization at more centers, or inadequate operator’s experience resulting in worse outcomes.^33^

Various known predictors of short-term mortality after MC are CKD, AF, >180 ​minutes of procedure time, disposition to a facility, elevated high-sensitive troponin T (≥75 pg/mL), reduced mixed venous oxygen saturation (<55%), and RV systolic dysfunction.^4^^,^^11^^,^^17^^,^^23^^,^^26^^,^^27^^,^^34^^,^^35^ Ischemic etiology of MR, previous coronary artery bypass grafting, and ejection fraction <30% were not associated with predicting survival of these patients.^20^^,^^22^^,^^35^ On the contrary, our study found no association of CKD and AF with early mortality, while LVEF was found to be a significant predictor.

One-year mortality after MC implantation has been reported to be 15.3%-20.3%^5^^,^^36^^,^^37^ or 18%-22% when estimated via predictive modeling.^38^ Age, advanced CHF, elevated pro-brain natriuretic peptide levels (>10,000 pg/mL), severe tricuspid regurgitation, dialysis, moderate or severe lung disease, and procedural factors such as “quality of repair” or residual MR are known to predict 1-year mortality.^4^^,^^10^^,^^20^ We did not find their association with our early mortality.

Previously, the incidence of 30-day CHF-related hospitalization after MC has been reported to be 4.9%.^4^ In our study, it was noted to be lower at 3.7%. Only 3.7% of our patients received MC after discharge as compared to the previously reported value of 6.2%.^4^ Functional MR patients had lower 30-day cumulative mortality than degenerative MR patients; however, this difference in mortality did not persist at 1 year.^6^^,^^21^ Explanations for this difference require further investigation.

Our study provides information about the risk and predictors of postdischarge-to-30-day mortality among MC patients. Certain patients carry an increased risk of dying immediately after discharge. Therefore, identifying and close monitoring are paramount for these high-risk patients to improve their outcomes. Further investigations and secondary data analysis of published clinical trials could provide better understanding of possible underlying mechanisms of deaths during this postprocedure period.

### Study Limitations

Our study has certain limitations. First, we have included retrospective observational studies, which may have their own inherent limitations due to study design. Second, we could not include detailed echocardiographic and other cardiac imaging parameters in our analysis. Third, heterogeneity of results was found to be high, which is attributed to the patient population receiving MC implantation in the real-world scenario. Fourth, postdischarge-to-30-day mortality among functional and degenerative MR was not calculated separately owing to the paucity of these specific data in the published literature. Fifth, deaths due to cardiac causes were reported only in 6 studies.^5^^,^^10^^,^^18^^,^^19^^,^^25^^,^^27^ Pooled data generated only very few events which could underestimate or overestimate the calculated proportion of 71.50% (as evident from 95% CI of 36.8%-91.5%). Our findings add important information to the current literature. Finally, we also evaluated within our included studies if surgery or reintervention could be considered as our early endpoint. However, it was not possible owing to lack of exact timing of MC procedure or surgical reintervention after the discharge.

## Conclusion

Our study found that high-risk surgical patients receiving MC experience ∼2% mortality in the postdischarge-to-30-day period. This forms a significant part of the cumulative 30-day mortality, with the majority of deaths occurring due to cardiac causes. LVEF, Log.Euroscore, STS PROM score, and prolonged ventilation were found to be predictors of postdischarge-to-30-day mortality. The procedural success rate was high. Few patients required in-hospital conversion to cardiac surgery or mitral valve reintervention during follow-up. Further studies are needed to better understand the causes of this early mortality which may provide an opportunity to maximize the benefits of this important therapy.

## Ethics Statement

Our study did not involve any direct interaction with human or animal subjects by any of the authors and thus did not meet criteria to require institutional review board approval.

## Funding

None of the authors received any funding for this project.

## Disclosure statement

The authors report no conflict of interest.
