# Supplementary material for: Postdischarge-to-30-Day Mortality Among Patients Receiving MitraClip: A Systematic Review and Meta-Analysis
Source: Struct Heart. 2022 Apr 26;6(1):100011. doi: 10.1016/j.shj.2022.100011 (PMC10236879; doi:10.1016/j.shj.2022.100011)
Supplement: Online Supplement 1 [file mmc1.pdf]

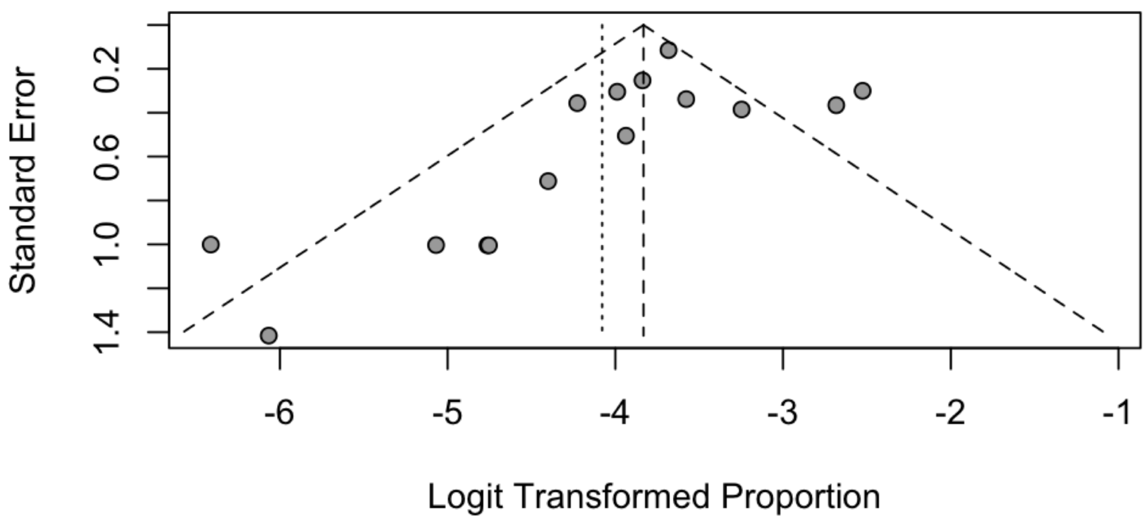

(a)

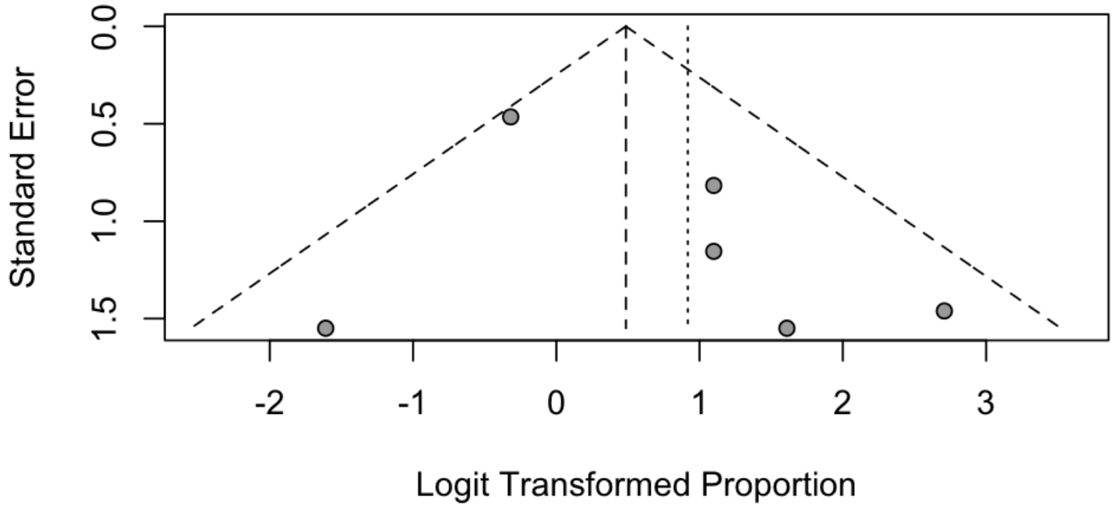

(b)

Online supplement 1: Funnel plot for visual assessment of small study effect a) all-cause post-discharge-to-30-days mortality – no small study effect (p=0.177) and b) post-discharge-to-30-days deaths due to cardiac causes– no small study effect
