# Supplementary material for: Postdischarge-to-30-Day Mortality Among Patients Receiving MitraClip: A Systematic Review and Meta-Analysis
Source: Struct Heart. 2022 Apr 26;6(1):100011. doi: 10.1016/j.shj.2022.100011 (PMC10236879; doi:10.1016/j.shj.2022.100011)
Supplement: Online Supplement 2 [file mmc2.pdf]

Author et al (year)	No. of deaths	Total patients	Proportion	95%-CI
Ailawadi et al (2019)	11	605	0.018	[0.009; 0.032]
Kitamura et al (2019)	9	331	0.027	[0.013; 0.051]
Kreusser et al (2019)	12	162	0.074	[0.039; 0.126]
Geis et al (2017)	16	759	0.021	[0.012; 0.034]
Osteresch et al (2018)	8	125	0.064	[0.028; 0.122]
Metze et al (2017)	4	209	0.019	[0.005; 0.048]
Saji et al (2017)	0	215	0.000	[0.000; 0.017]
Giannini et al (2016)	2	165	0.012	[0.001; 0.043]
Oner et al (2017)	1	160	0.006	[0.000; 0.034]
Schau et al (2016)	7	187	0.037	[0.015; 0.076]
Bozdag-Turan et al (2014)	1	118	0.008	[0.000; 0.046]
Nickenig et al (2014)	1	610	0.002	[0.000; 0.009]
Grasso et al (2013)	1	117	0.009	[0.000; 0.047]
Maisano et al (2013)	8	556	0.014	[0.006; 0.028]
**Fixed effect model**		**4319**	**0.019**	**[0.015; 0.023]**
**Random effects model**			**0.016**	**[0.009; 0.027]**
**Prediction interval**				**[0.002; 0.106]**

Heterogeneity: I^2 = 80%, τ^2 = 0.7683, p < 0.01

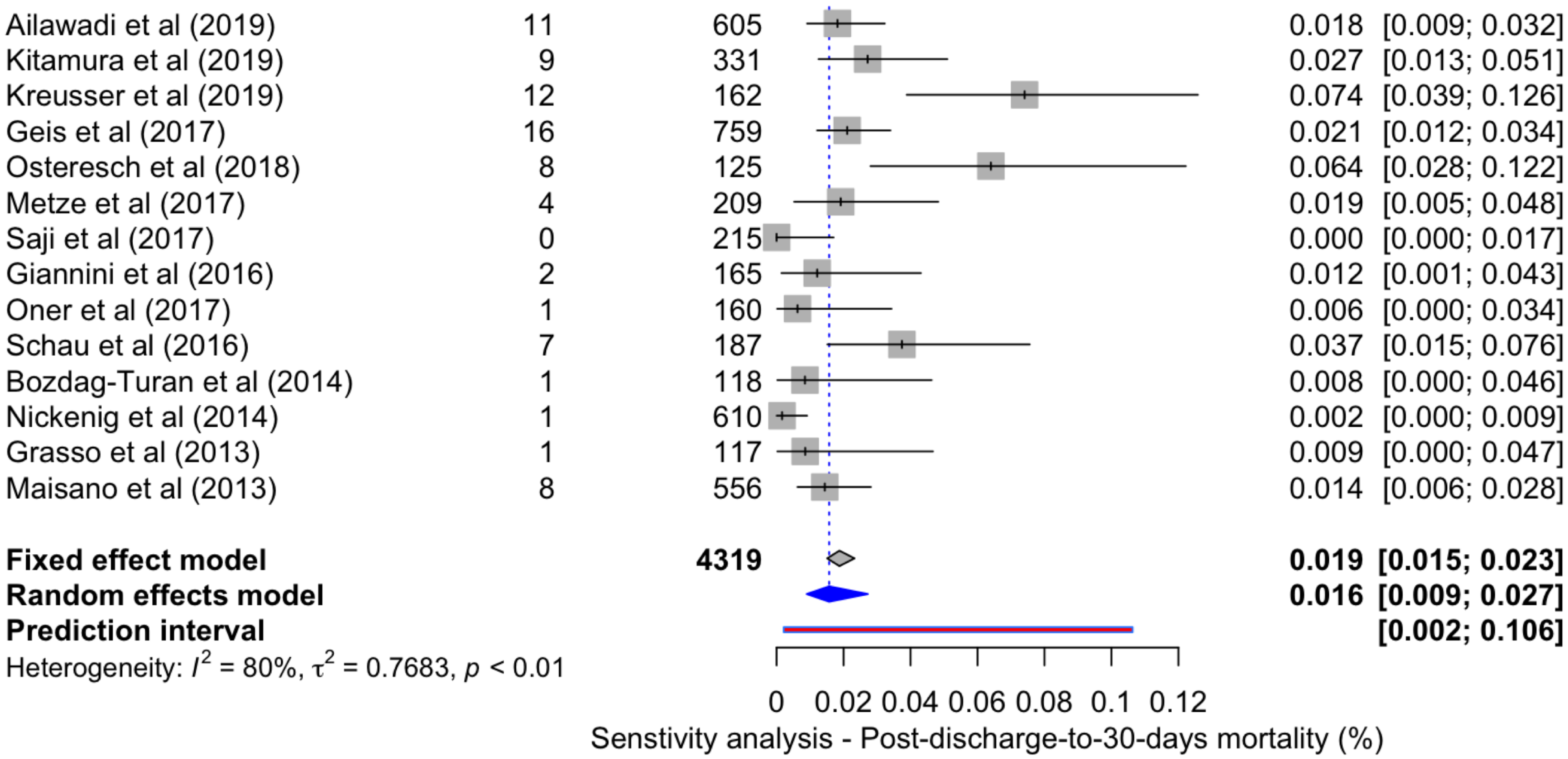

Online supplement 2: Forest plot showing individual and summary proportion of all cause post-discharge-to-30-days mortality (sensitivity analysis) as 1.60% [95% CI: 0.90%-2.7%, I^2=80%]
