# Supplementary material for: Postdischarge-to-30-Day Mortality Among Patients Receiving MitraClip: A Systematic Review and Meta-Analysis
Source: Struct Heart. 2022 Apr 26;6(1):100011. doi: 10.1016/j.shj.2022.100011 (PMC10236879; doi:10.1016/j.shj.2022.100011)
Supplement: Appendix A [file mmc3.pdf]

| Author/Year of publication | Selection | | | | Comparability | Outcome | | | Total no. of stars |
|---|---|---|---|---|---|---|---|---|---|
| | S1 | S2 | S3 | S4 | C | O1 | O2 | O3 | |
| Ailawadi et al (2019) | ★ | NA | ★ | ★ | NA | ★ | ★ | ★ | 6 |
| Arora et al (2019) | ★ | NA | ★ | ★ | NA | ★ | ★ | ★ | 6 |
| Kitamura et al (2019) | ★ | NA | ★ | ★ | NA | ★ | ★ | ★ | 6 |
| Kreusser et al (2019) | ★ | NA | ★ | ★ | NA | ★ | ★ | ★ | 6 |
| Geis et al (2017) | ★ | NA | ★ | ★ | NA | ★ | ★ | ★ | 6 |
| Osteresch et al (2018) | ★ | NA | ★ | ★ | NA | ★ | ★ | ★ | 6 |
| Metze et al (2017) | ★ | NA | ★ | ★ | NA | ★ | ★ | ★ | 6 |
| Saji et al (2017) | ★ | NA | ★ | ★ | NA | ★ | ★ | ★ | 6 |
| Giannini et al (2016) | ★ | NA | ★ | ★ | NA | ★ | ★ | ★ | 6 |
| Oner et al (2016) | ★ | NA | ★ | ★ | NA | ★ | ★ | ★ | 6 |
| Schau et al (2016) | ★ | NA | ★ | ★ | NA | ★ | ★ | ★ | 6 |
| Bozdag-Turan et al (2014) | ★ | NA | ★ | ★ | NA | ★ | ★ | ★ | 6 |
| Nickenig et al (2014) | ★ | NA | ★ | ★ | NA | ★ | ★ | ★ | 6 |
| Grasso et al (2013) | ★ | NA | ★ | ★ | NA | ★ | ★ | ★ | 6 |
| Maisano et al (2013) | ★ | NA | ★ | ★ | NA | ★ | ★ | ★ | 6 |

**Appendix A**: Newcastle and Ottawa Scale: Evaluating quality of the evidence
