# Supplementary material for: Postdischarge-to-30-Day Mortality Among Patients Receiving MitraClip: A Systematic Review and Meta-Analysis
Source: Struct Heart. 2022 Apr 26;6(1):100011. doi: 10.1016/j.shj.2022.100011 (PMC10236879; doi:10.1016/j.shj.2022.100011)

**Appendix B**: Association of study level characteristics with post-discharge-to-30-days mortality

(Metargression was performed between post-discharge-to-30-days mortality as our primary outcome with study level characteristics as age, male gender, hypertension, cerebrovascular accidents, diabetes mellitus, chronic obstructive lung disease, body mass index (kg/m2), coronary artery disease, myocardial infarction, coronary artery bypass grafting, atrial fibrillation, congestive heart failure, renal failure, peripheral artery disease, single vs multicenter, procedural success, post MC MR>3, pre-MC severe TR, Number of mitral valve clips>1, clip detachment, post MC bleeding and post MC conversion to open-heart surgery. MC: MitraClip; MR: mitral regurgitation; TR: tricuspid regurgitation)


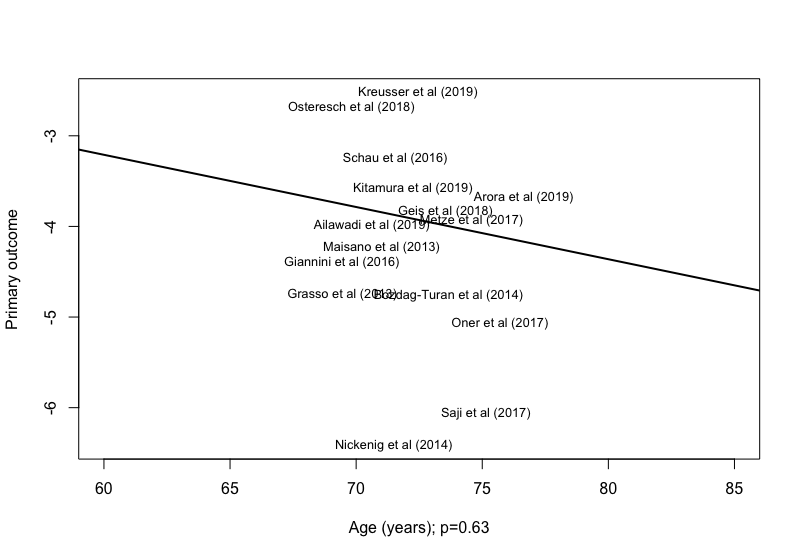

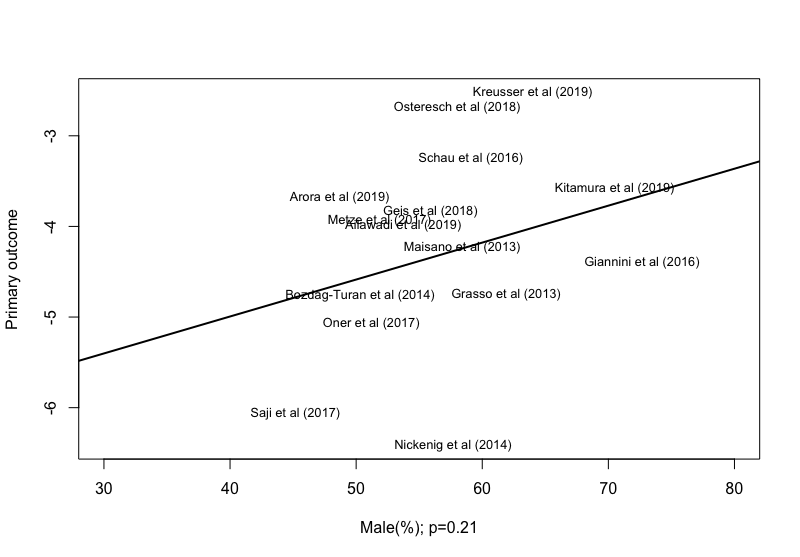

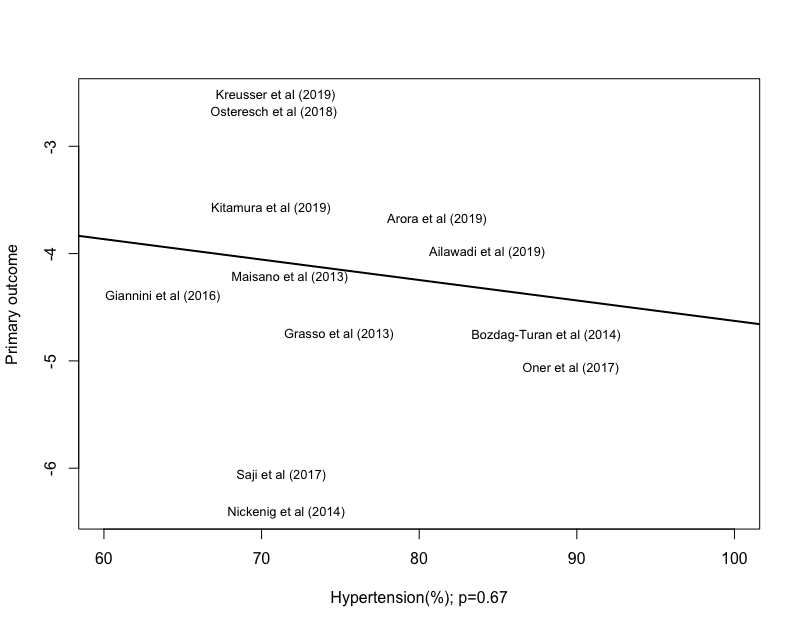

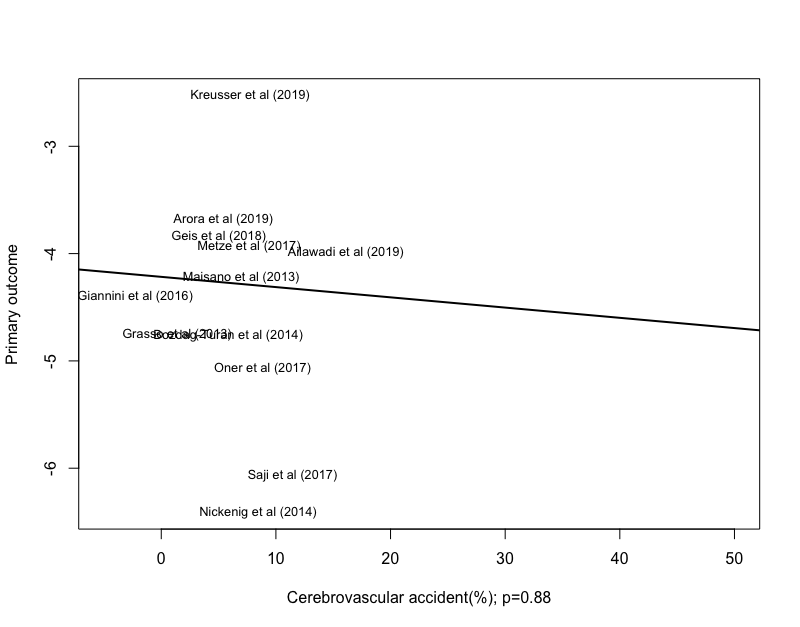

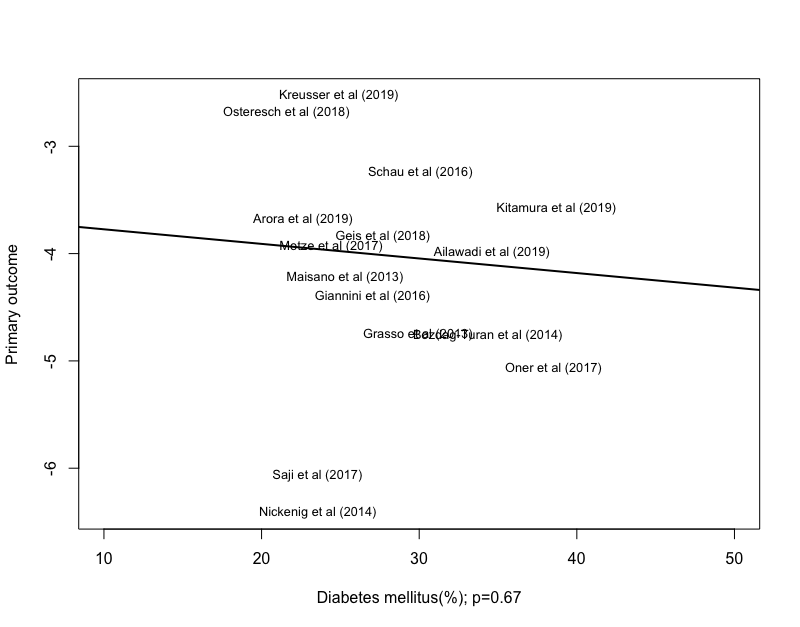

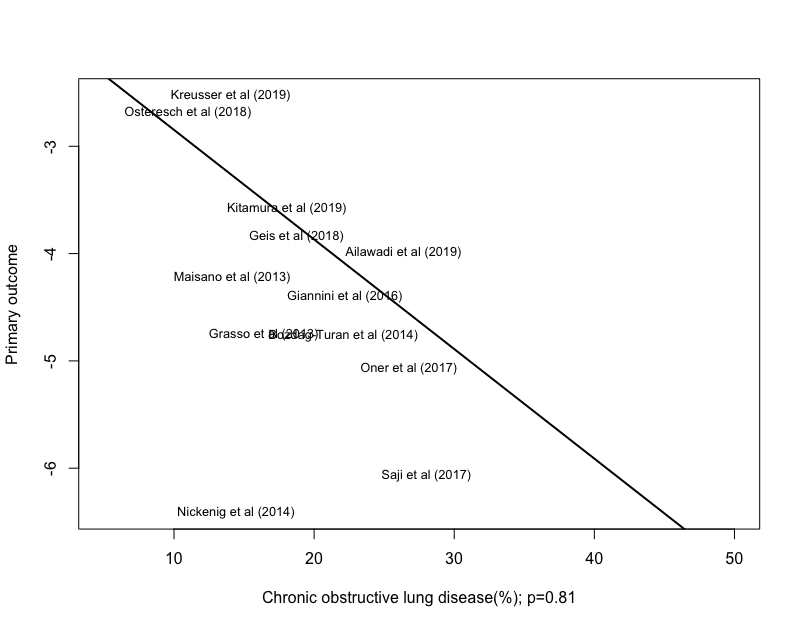

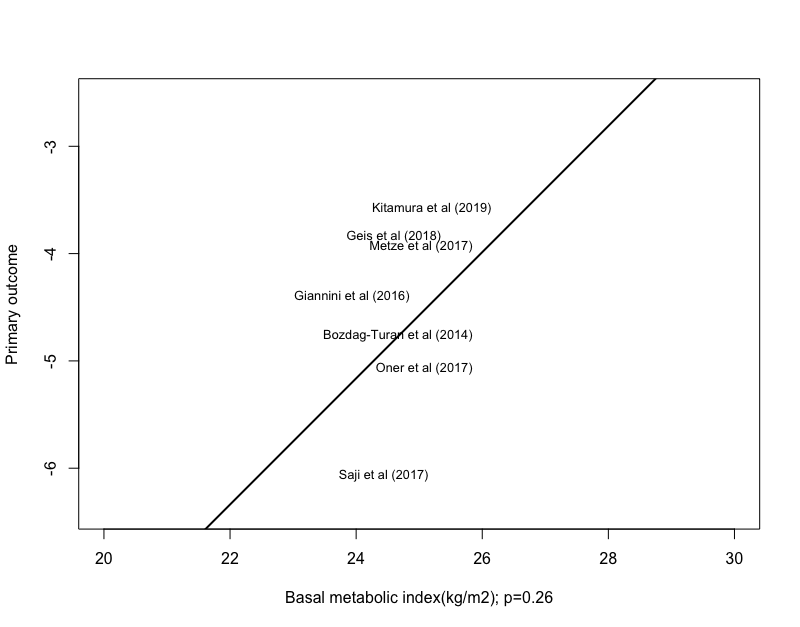

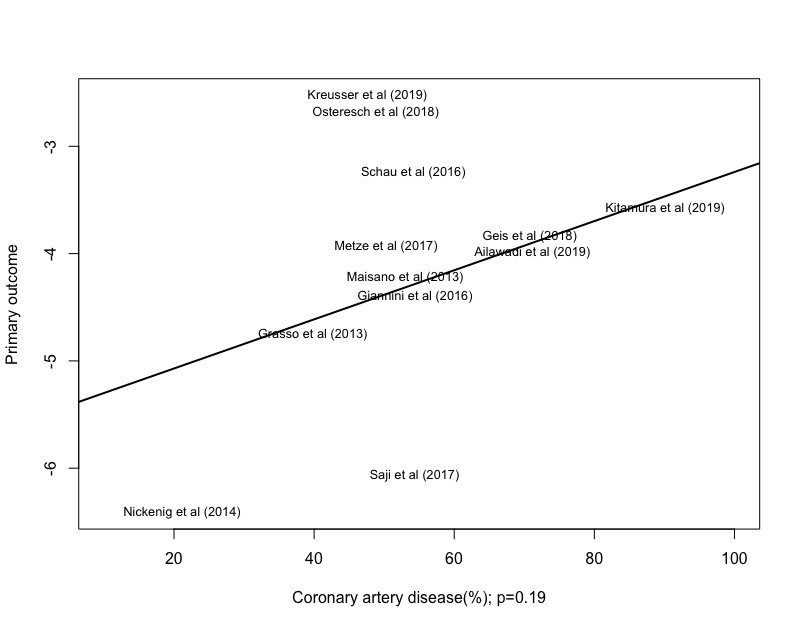

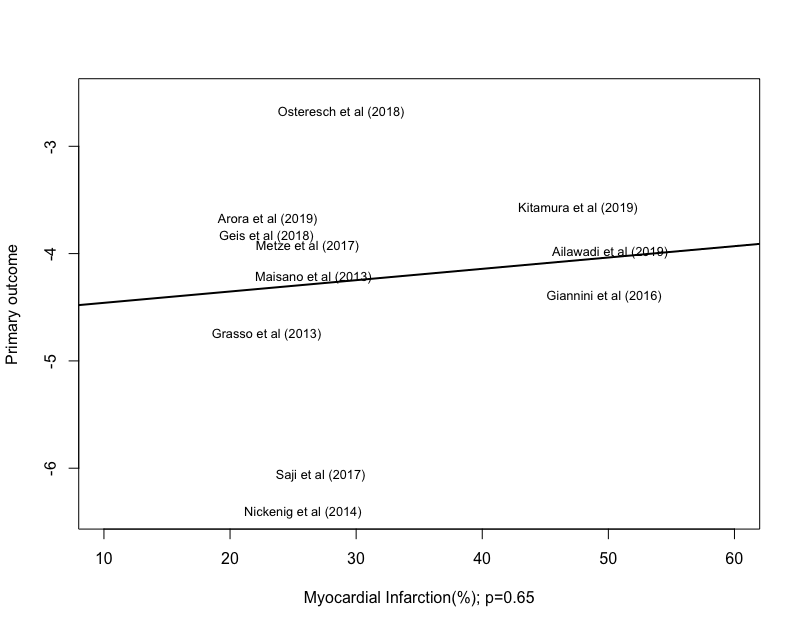

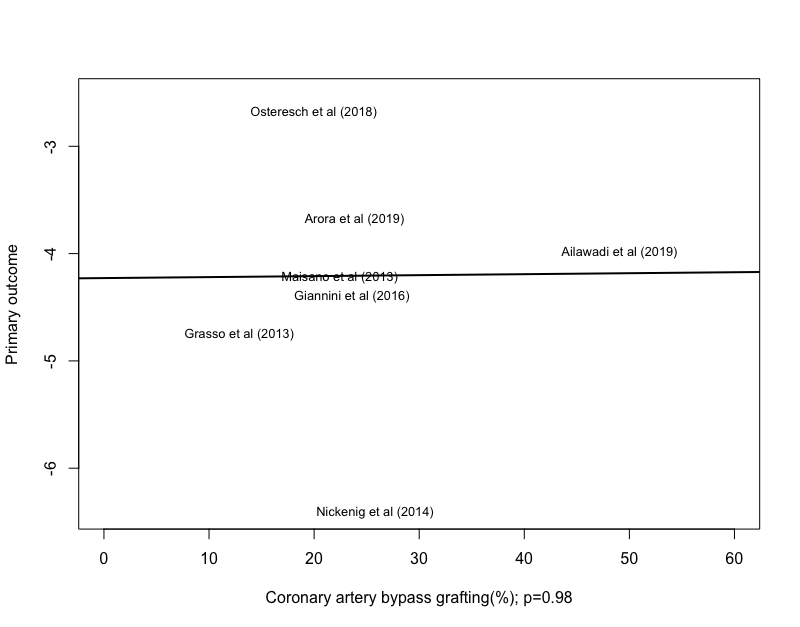

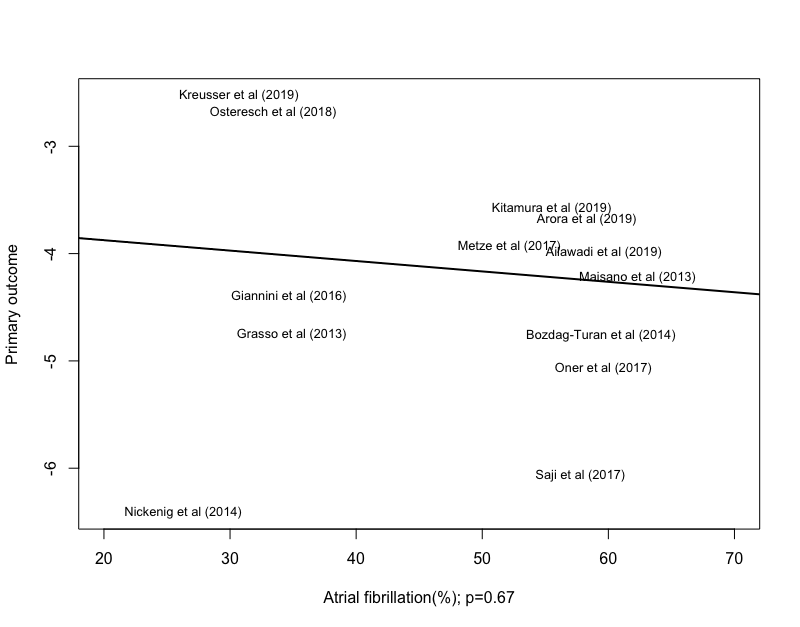

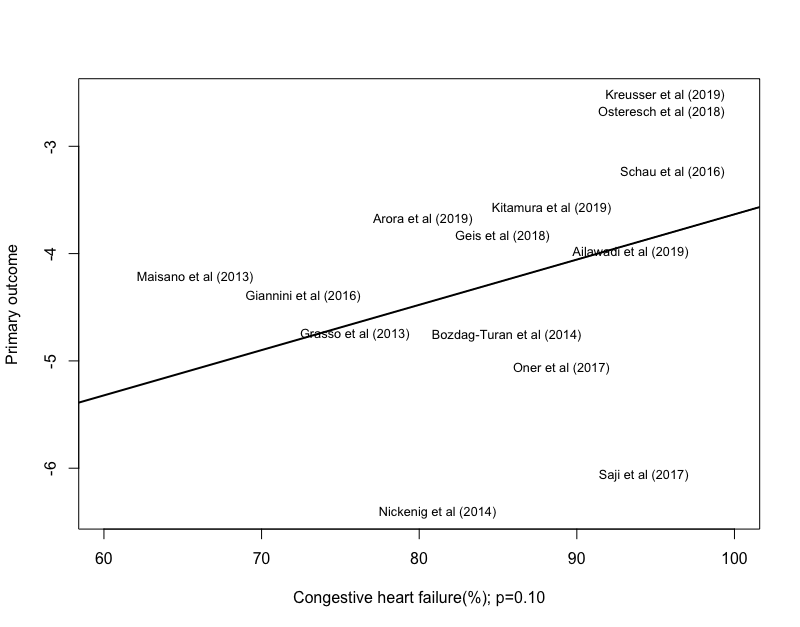


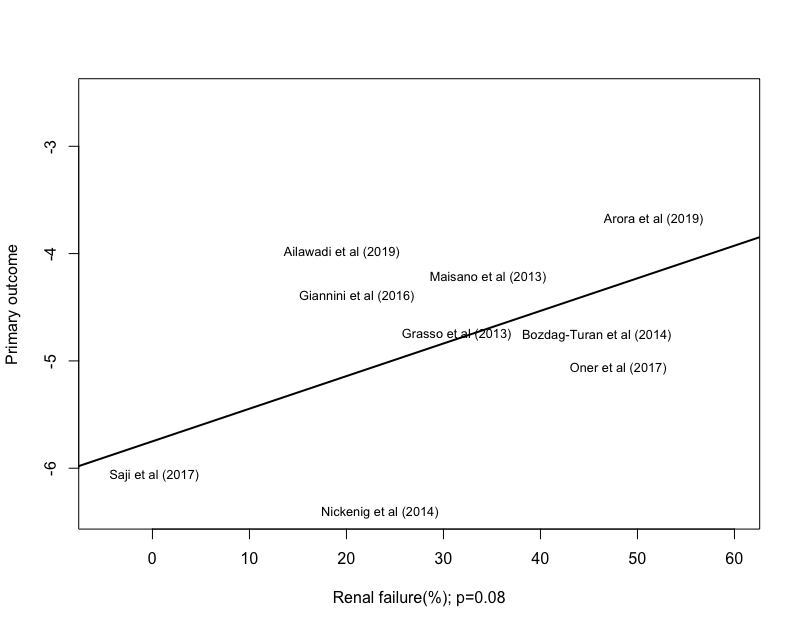


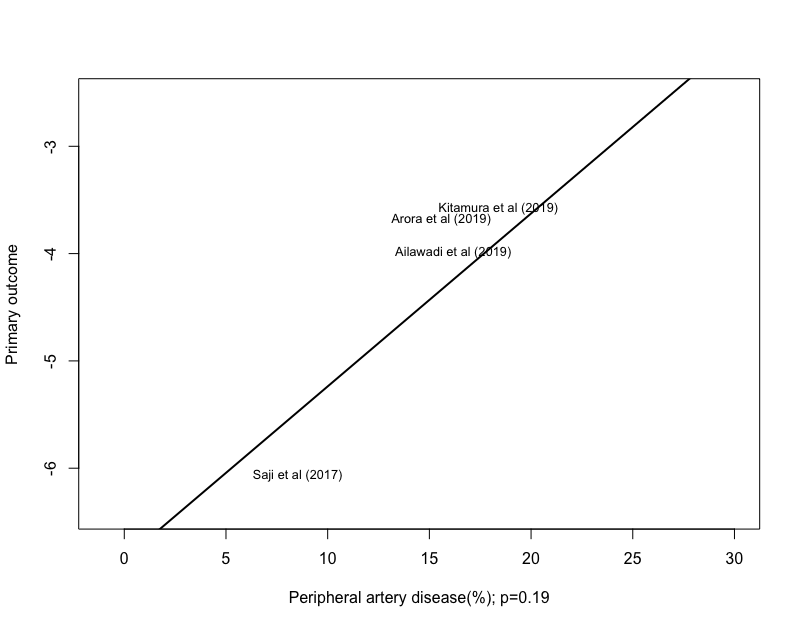


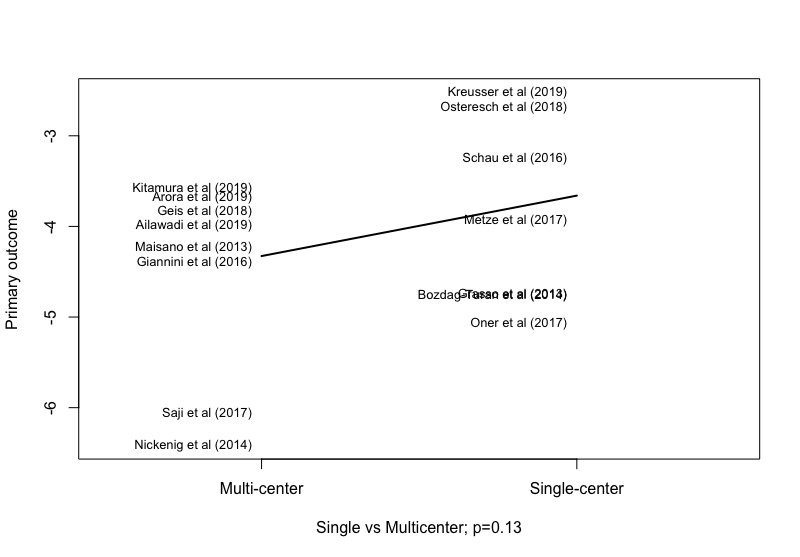


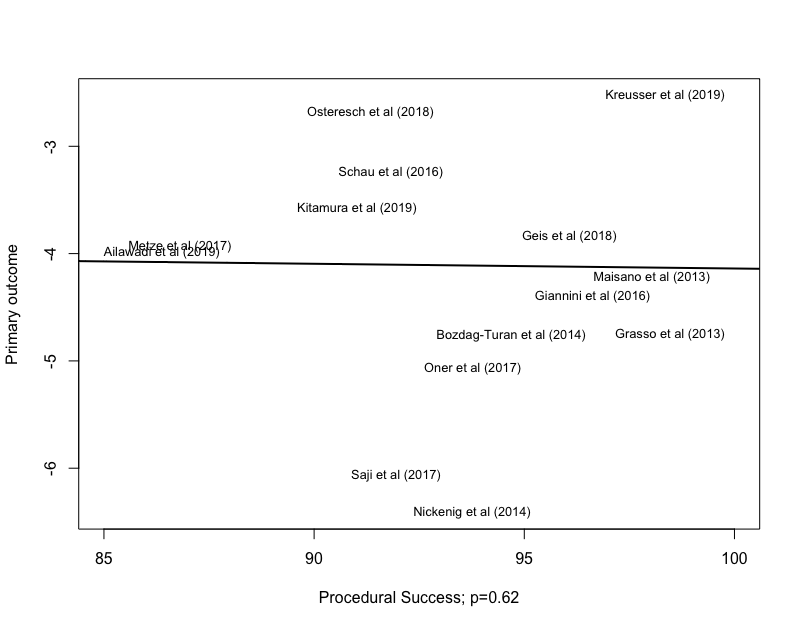


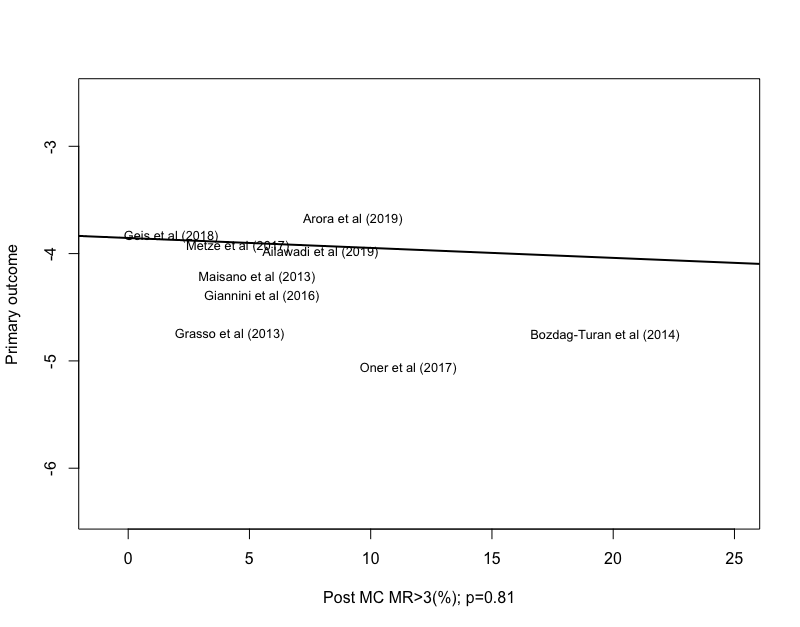


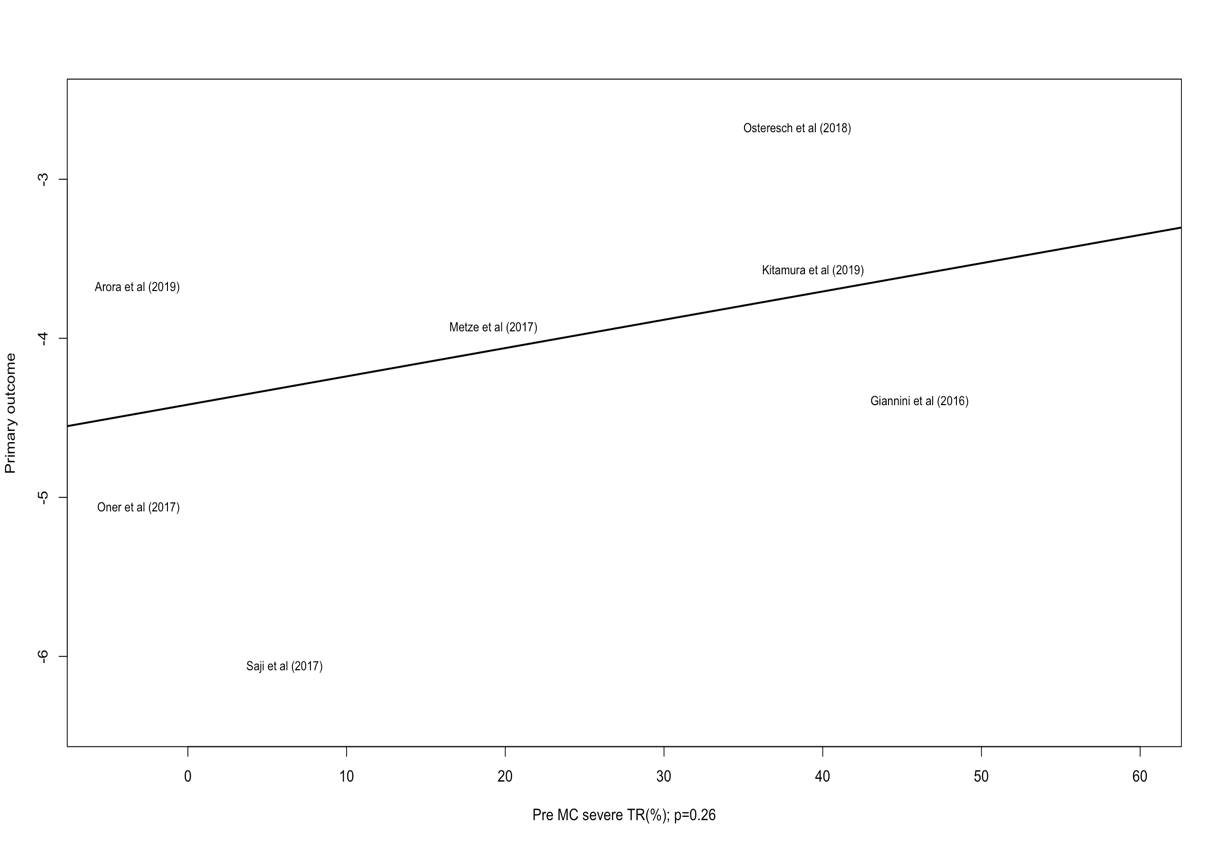


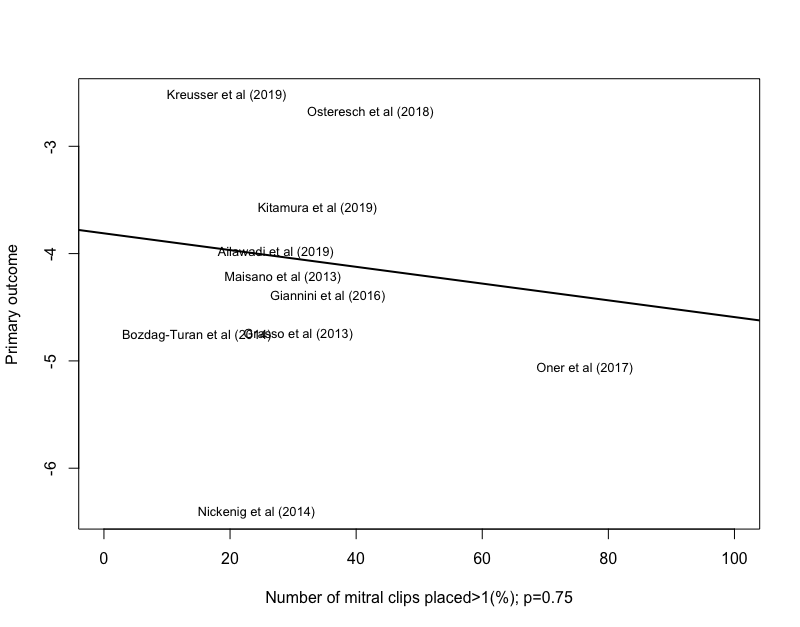


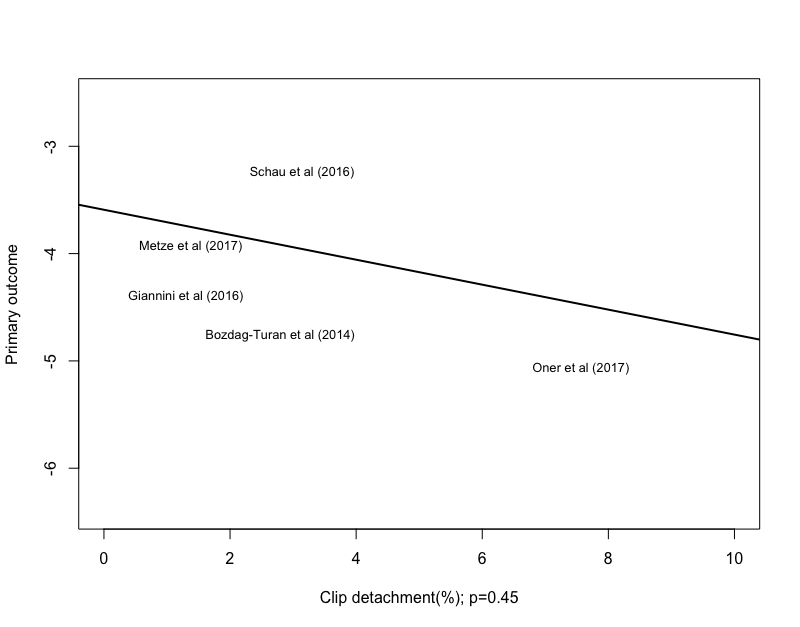


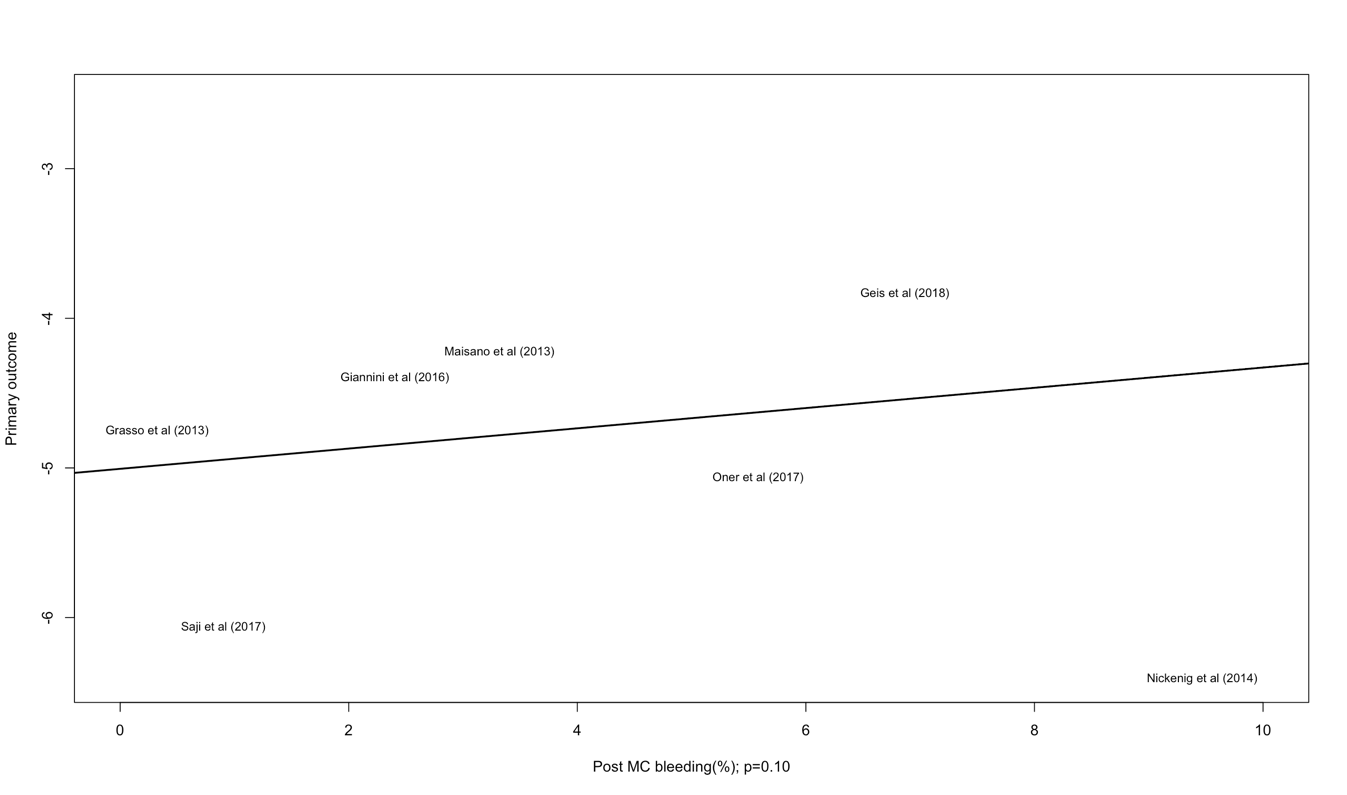


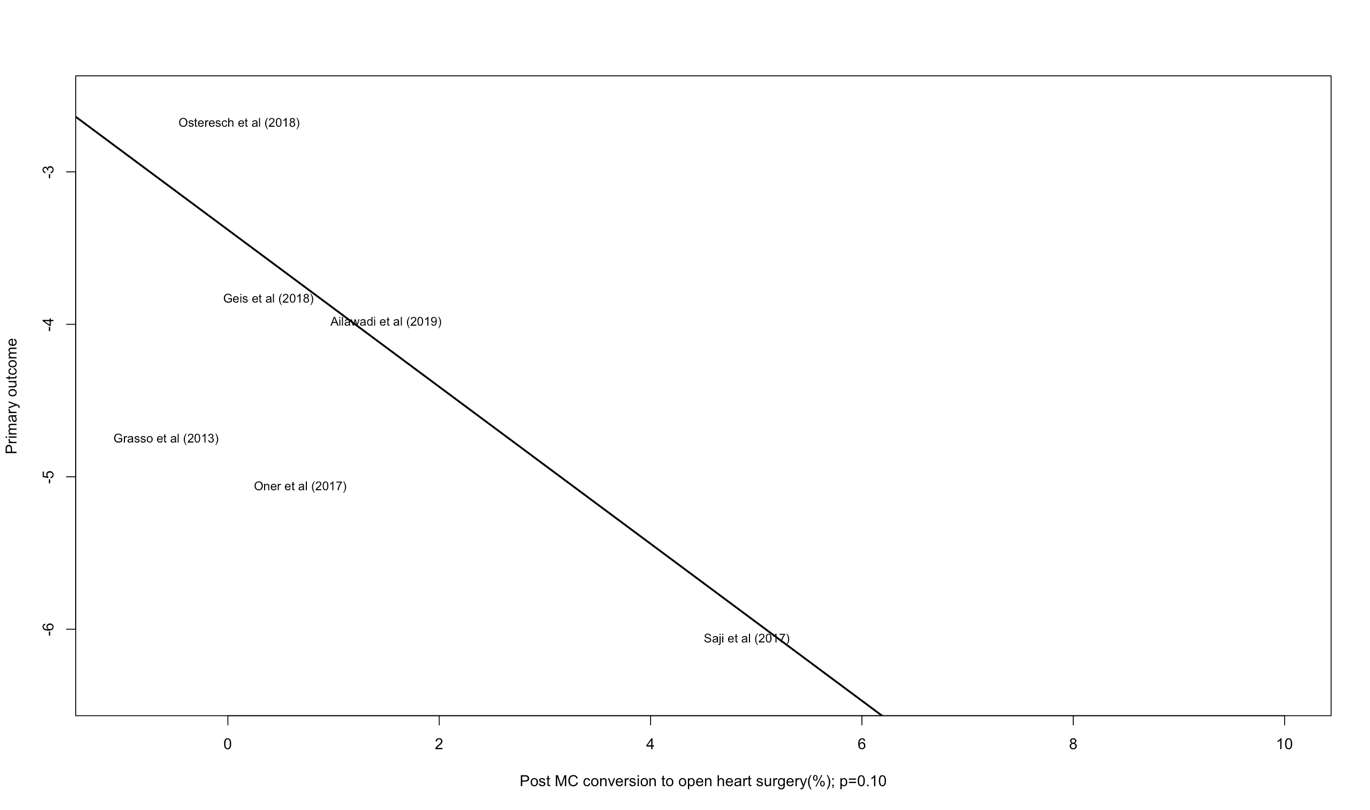

Supplement: Appendix B [file mmc4.docx]
